# Reprogrammable CRISPR/dCas9-based recruitment of DNMT1 for site-specific DNA demethylation and gene regulation

**DOI:** 10.1038/s41421-019-0090-1

**Published:** 2019-04-16

**Authors:** Anrui Lu, Jingman Wang, Weihong Sun, Weiren Huang, Zhiming Cai, Guoping Zhao, Jin Wang

**Affiliations:** 1 Carson International Cancer Center, Shenzhen University School of Medicine, Shenzhen Second People’s Hospital, The First Affiliated Hospital of Shenzhen University, Shenzhen, 518039 China; 2 The State Key Laboratory of Oncology in South China, Sun Yat-Sen University Cancer Center, Guangzhou, 510060 China; 3 Shanghai Institute of Nutrition and Health, Shanghai Institutes for Biological Sciences, Chinese Academy of Sciences, Shanghai, 200032 China; 4 Guangdong Key Laboratory of Systems Biology and Synthetic Biology, Urogenital Tumors, Shenzhen, 518035 China; 5 Key Laboratory of Synthetic Biology, Institute of Plant Physiology and Ecology, Shanghai Institutes for Biological Sciences, Chinese Academy of Sciences, Shanghai, 200032 China; 6 College of Life and Environment Sciences, Shanghai Normal University, Shanghai, 200234 China

**Keywords:** Methylation analysis, DNA methylation

Dear Editor,

DNA methylation and demethylation play a critical role in regulating gene expression as well as developmental and pathological processes^1^. For example, hyper-methylation in the promoter region typically represses a gene’s transcription, and aberrant DNA methylation has been demonstrated to be associated with certain tumors^2^. Till now, many enzymes have been demonstrated to be involved in DNA methylation or demethylation, including DNA methyltransferases, which use S-Adenosyl methionine as the methyl donor to catalyze the transfer of a methyl group to target DNA^1^. In mammalian cells, three active DNA methyltransferases have been characterized, including DNMT1, DNMT3A, and DNMT3B^1^. DNMT1, the most abundant methyltransferase, predominately methylates cytosines at hemi-methylated CpG dinucleotides^1,3^ and is responsible for the maintenance of DNA methylation, ensuring the fidelity of inherited epigenetic patterns during replication. To perform loci-specific demethylation modification in vivo, both Zinc fingers (ZFs)^4^ and transcription activator-like effectors (TALEs)^5^ have been used to fuse the ten-eleven translocation methylcytosine dioxygenase 1 (TET1) catalytic domain for DNA demethylation. However, both the design and the protein engineering of the ZFs (or TALEs) that are required for the binding of specific loci are time-consuming and costly, and their applications are therefore limited. Recently, with the improvement of the CRISPR (clustered regularly interspaced short palindromic repeats) technology, catalytically dead Cas9 (dCas9) was fused to the catalytic domain of TET1 (TET1CD) to hydroxylate specific loci and activate site-specific demethylation. Although this CRISPR system brings much convenience, it requires expression of a foreign TET1 enzyme^6^, and may therefore cause unexpected influences on cellular metabolisms. To minimize the introduction of foreign components, we here develop a new CRISPR/dCas9-based system (CRISPR/dCas9-R2) to reduce the DNA methylation level by blocking the DNMT1 activity in the targeted loci (Fig. 1a). Specifically, a short RNA sequence with the stem-loop structure (named R2-stemloop, Fig. 1b) is introduced to both positions of the tetraloop and the stemloop2 of the single-guide RNA (sgRNA) scaffold. Then, DNMT1 is specifically recruited by the R2-stemloop and its enzyme activity is inhibited upon binding to the R2-stemloop structure, therefore the methylation of the targeted DNA loci is blocked^7^.

**Fig. 1:**
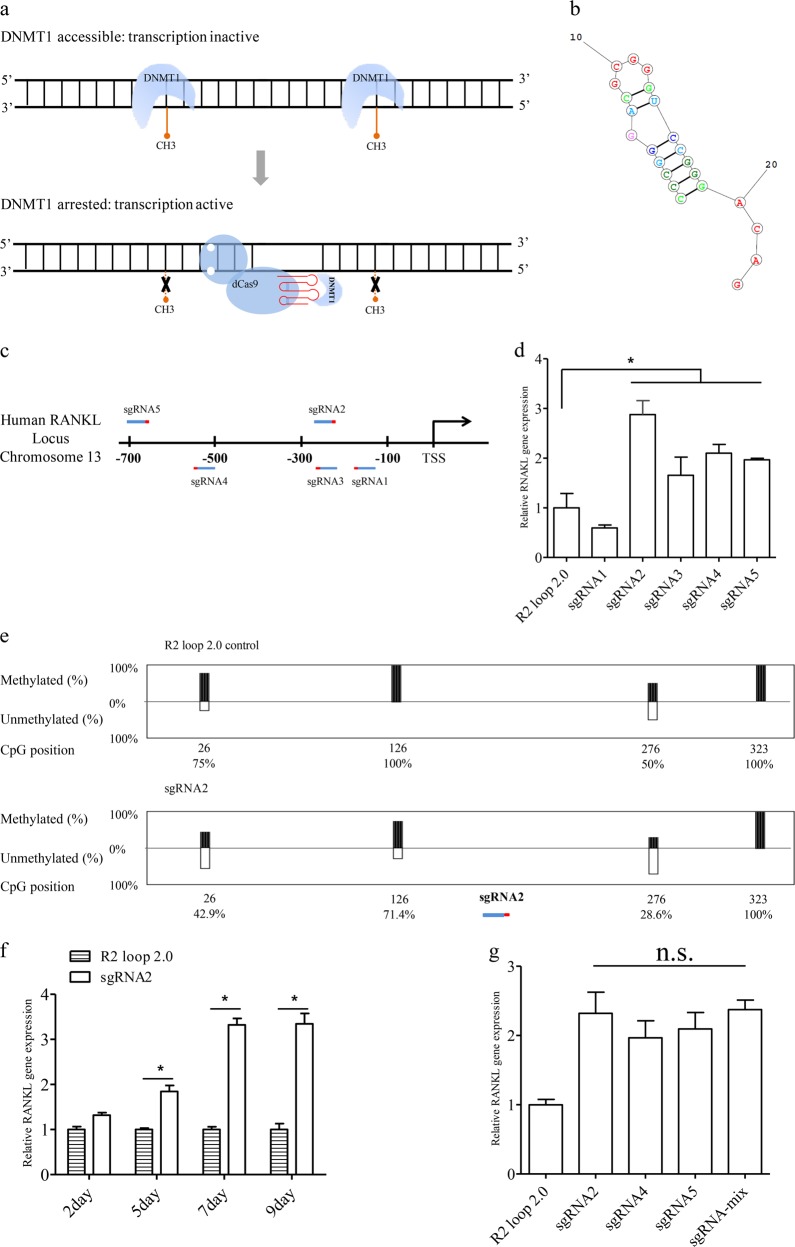
CRISPR/dCas9-R2 based DNA demethylation system. **a** Schematic description of demethylation of targeted DNA sequences by restricting the DNMT1 activity. The DNMT1 is recruited by the sgRNA-R2 stemloop, and the methyltransferase activity is therefore inhibited, resulting in the decrease of the DNA methylation level. **b** The RNA structure of the R2 stemloop. **c** Illustration of the five sgRNAs designed for targeting the promoter region of the human *RANKL* gene. The sgRNAs were shown in blue-red color, where the blue color represented the guide sequences and the red color represented the PAM region, and the sgRNAs located at about 700 bps upstream of the transcriptional start site of *RANKL*. **d** Determination of the abundances of *RANKL* mRNA in HEK-293T cells by quantitative RT-PCR assay. **e** Measurement of the percentages of methylated and unmethylated DNA in the *RANKL* promoter region by the bisulfate sequencing method. The presented data were first normalized to the reference group (i.e., the CRISPR/dCas9-R2 system using an sgRNA with no guide sequence), and the accurate value of methylation level was shown below each CpG site. **f** Assessment of the transcriptional level of *RANKL* gene by quantitative RT-PCR assay. Total RNA was prepared at the different post-transfection days from the HEK-293T cells (i.e., 2–9 days). **g** Determination of the additive effect of the CRISPR/dCas9-R2 system in upregulating gene expression by quantitative RT-PCR assay. Either individual sgRNA (i.e., RANKL sgRNA-2, 4 and 5) or a mixture of these sgRNAs resulted in a similar level of transcriptional activation

The human *RANKL* gene has been reported to be repressed because of hyper-methylation in its promoter region in HEK-293T cells^8^, and demethylation in its promoter region could potentially activate the gene expression. To test whether the transcription of *RANKL* could be regulated by the CRISPR/dCas9-R2 system, five sgRNAs were designed to target the *RANKL* promoter region (Fig. 1c), and the impact of individual sgRNA on *RANKL* transcription was assayed by quantitative RT-PCR (qRT-PCR), using total RNA extraction prepared from the HEK-293T cells 5 days post transfection. Three of the five sgRNAs (i.e., RANKL-sgRNA2, 4, 5) were found to be able to activate the transcription of *RANKL* (Fig. 1d), among which RNAKL-sgRNA2 was the most effective. We then used the bisulfite-sequencing method to examine the methylation status of four CpG sites in the RANKL-sgRNA2 targeted locus, and found three of them (i.e., positions of 26, 126, and 276) had a decreased methylation level (Fig. 1e and Supplementary Table S1), indicating the close relationship between the upregulation of *RANKL* transcription and decreased methylation status in the promoter region. In addition, we also used the CRISPR/dCas9-R2 system to regulate the transcription of another gene (namely *MAGEB2*), which locates on the chromosome X and is also transcriptionally repressed by DNA methylation in its promoter region^9^. Similarly, five sgRNAs were designed to target the promoter region of *MAGEB2* (Supplementary Fig. S1a), and four of them (i.e., MAGEB2-sgRNA2, 3, 4 and 5) showed effectiveness in upregulating *MAGEB2* transcription (Supplementary Fig. S1b). In accordance with the transcriptional activation, the DNA methylation status in *MAGEB2* promoter region was found to be decreased in cells transfected with MAGEB2-sgRNA2 and 3. Specifically, four of the eight CpG sites (i.e., positions of 119, 134, 189, and 257) had a decreased methylation level (Supplementary Fig. S1c and Supplementary Table S2).

We next examined the influence of the transfection time on demethylation efficiency by the CRISPR/dCas9-R2 system, and the *RANKL* transcription in HEK-293T cells was analyzed by qRT-PCR at different time points after transfection of RNAKL-sgRNA2. As shown in Fig. 1f, the transcription level of *RANKL* reached the highest at the 7th day post transfection (i.e., about threefold relative to the control group with no-sgRNA transfected). In accordance with the transcriptional results, three methylated CpG sites in *RANKL* promoter region (i.e., positions of 26, 126, and 276) also decreased gradually, and reached the lowest level at the 7th day (Supplementary Table S3). Similarly, transcriptional activation of *MAGEB2* also became the most effective at the 7th day post transfection (Supplementary Fig. S1d). Moreover, we checked whether there existed additive effects on upregulating target gene expression by transfecting more than one sgRNAs targeting to the promoter region. For target *RANKL* gene, either individual or mixture of the three effective sgRNAs (i.e., RANKL-sgRNA2, 4 and 5) was transfected into the HEK-293T cell, and the transcription of *RANKL* was analyzed 5 days post transfection. According to the qRT-PCR results (Fig. 1g), either individual or mixture of sgRNAs led to similar level of transcriptional activation. Similarly, transfection of an sgRNA mixture did not lead to a higher transcriptional level of *MAGEB2* (Supplementary Fig. S1e), therefore indicating the dCas9-R2 system had no additive effects for gene activation.

Here, we described a programmable approach for convenient demethylation of targeted DNA sequences in living cells. Considering the availability of multiple Cas9 mutants recognizing different PAM sites^10^, the CRISPR/dCas9-R2 system using either dCas9 or other dCas9 mutants may have the potential to target any DNA sequences. Compared with currently available CRISPR-based approaches for targeting DNA demethylation, our system showed similar editing efficiencies with the CRISPR/dCas9-TET1 system^11^ but does not require co-transfection of other epigenetic enzymes, therefore minimizing unexpected influences on cellular metabolisms. Besides, the editing window of the CRISPR/dCas9-R2 system is about 100-bp distance nearby the targeted site (Fig. 1e, Supplementary Fig. S1c and Supplementary Tables S1– S3), and could be more accurate than the present CRISPR/dCas9-TET1 system^11^, which is effective in regions of 100–300-bp distance from the targeted site. Moreover, advantage of CRISPR/dCas9-R2 system compared with other methods such as ZFs^4^ and TALEs^5^ which are challenging in the system construction is also summarized (Supplementary Table S4).

## Supplementary information

Supplementary Information

## Supplementary information

**Supplementary Information** accompanies the paper at (10.1038/s41421-019-0090-1).
