# Supplementary material for: Reprogrammable CRISPR/dCas9-based recruitment of DNMT1 for site-specific DNA demethylation and gene regulation
Source: Cell Discov. 2019 Apr 16;5:22. doi: 10.1038/s41421-019-0090-1 (PMC6465405; doi:10.1038/s41421-019-0090-1)
Supplement: Supplementary file 1 — Supplementary Information [file 41421_2019_90_MOESM1_ESM.pdf]

Materials and Methods

Plasmids construction

The CRISPR/dCas9-R2 plasmid was constructed based on plasmid pSpCas9(BB)-2A-Puro (PX459)[1]. SpCas9 was subject to D10A and H840A substitution to construct dSpCas9. The R2 stemloop RNA were inserted into U6 promoter driven sgRNA cassette at the tracrRNA tetra stemloop and stemloop2 position. All the designed were synthesized as oligos (Biosune, Shanghai, China), annealed and cloned into CRISPR/dCas9-R2 at BpiI (Thermo Scientific, Shanghai, China) digestion site. Sequences of sgRNA target sites used in the construction of CRISPR/dCas9-R2 expression plasmids were listed in Supplementary Table S6.

HEK-293T cell culture and transfection

HEK-293T (Cell bank, Shanghai Institutes for Biological Science, Shanghai, China) was cultured in DMEM medium (HyClone, South logan, UT, USA) with 10% fetal bovine serum (Tianhang, Hangzhou, China) and penicillin/streptomycin (Biosharp, Hefei, China). Cells were incubated in a humidified 5% CO_2 air incubator at 37°C. Cells were seeded in 6-well cell culture plate (Corning, NY, USA) before transfection about 1 day. Different plasmids were transfected into HEK-293T used Attractene Transfection Reagent (QIAGEN, Duesseldorf, Germany) according to the manufacturer's protocols. Cells were harvested at different time point post-transfection.

qRT-PCR

Total RNAs were isolated from cells of indicated groups using Trizol Reagent (Invitrogen, Carlsbad, CA, USA). Complementary DNA was synthesized using PrimeScript™ RT reagent Kit with gDNA Eraser (Perfect Real Time) (TaKaRa, Dalian, China) according to the manufacturer's protocol. qRT-PCR assay was performed to assess the relative abundances of the mRNA, using specific primers of sequences listed in Supplementary Table S5, stained SYBR Green (TaKaRa, Dalian, China) on ABI Step one plus real-time PCR system (Applied Biosystems) to examine gene transcription. The relative abundances of the transcripts of indicated genes were normalized to that of GAPDH, using $\Delta\Delta$Ct method. All data were obtained from at least three independent experiments.

Bisulfite DNA sequencing

Genomic DNA was extracted from cells of indicated groups using the cell genome extraction kit (Tiangen, Beijing, China) according to the manufacturer's protocol. Genomic DNA was treated with bisulfite using EZ DNA Methylation™ Kit (Zymo Research, Irvine, California, USA) according to the manufacturer's manual. The modified DNA was amplified using EsayTaq DNA polymerase (TransGen, Beijing, China) the respective primer sets that recognize bisulfate-modified DNA only (Primer sequences listed in Supplementary Table S5). Then the PCR products were cloned into pMD-19T vector (TaKaRa, Dalian, China), followed by Sanger sequencing.

Statistics

Statistical analyses were performed with unpaired T-test using GraphPad Prism (version 5 for Windows, GraphPad Software, San Diego, CA, USA).

Supplementary Figures

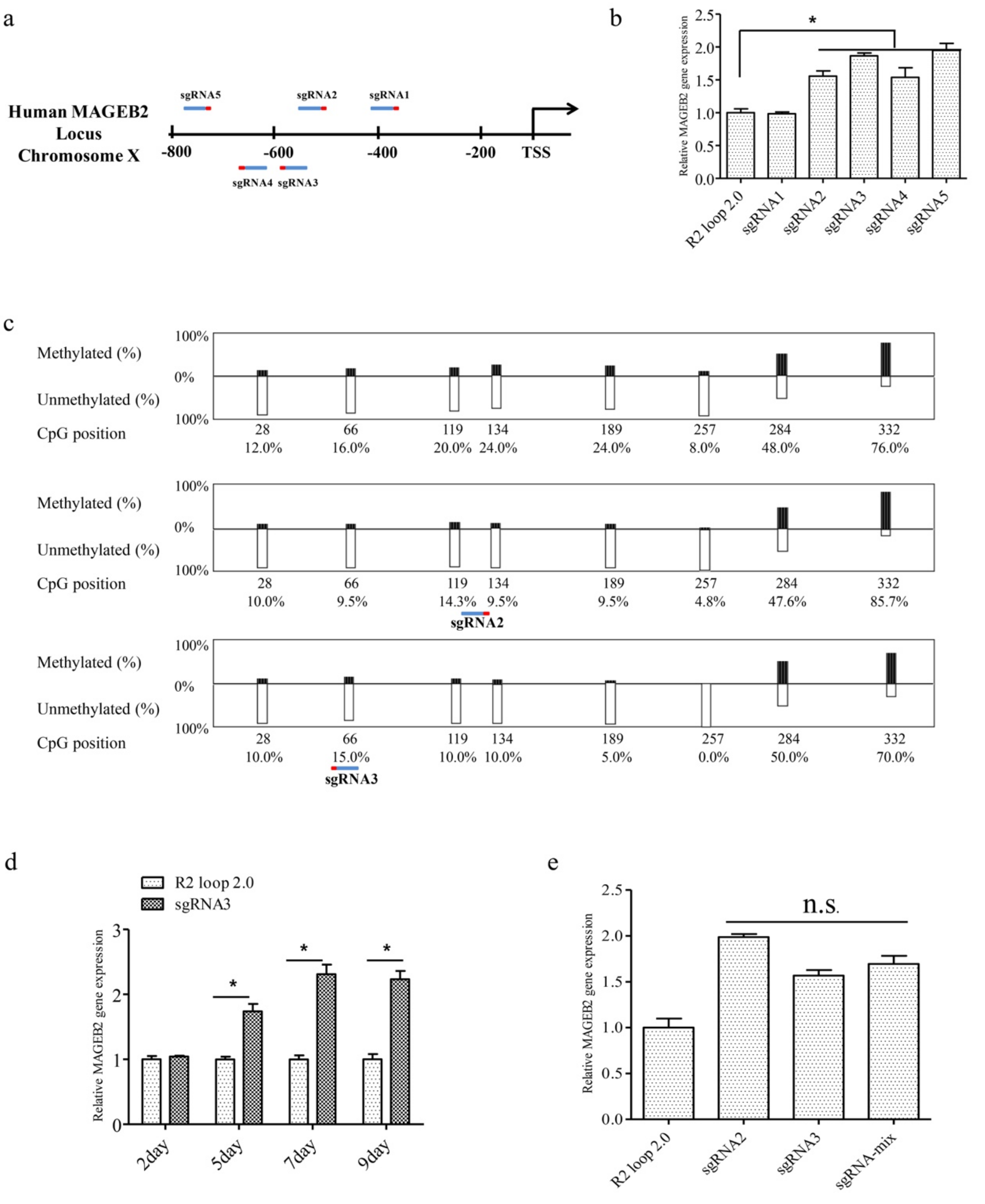

Supplementary Figure S1. (a) Five sgRNAs were selected targeting chromosome regions within about 800 bps upstream to the transcriptional start site of human *MAGEB2* gene. The sgRNAs recognized their specific sites were shown in blue-red color, where the blue color represented the guide sequences and the red color represented the PAM region. (b) The abundances of *MAGEB2* mRNA in HEK-293T cells determined by quantitative RT-PCR assay. (c) The percentages of methylated or unmethylated DNA in *MAGEB2* gene promoter region, assayed by bisulfate sequencing. Data were shown after normalization to the control group (CRISPR/dCas9-R2 system without guide sequence), and the accurate values were presented under each site. (d) Assessment of the transcriptional level of

MAGEB2 gene by qRT-PCR assay, using total RNA prepared at the different days post transfection of the HEK-293T cells (i.e. 2 - 9 days). (e) Determination of the additive effect of the CRISPR/dCas9-R2 system in up-regulating *MAGEB2* expression by quantitative RT-PCR assay. Either individual sgRNA (i.e. MAGEB2 sgRNA-2, 3) or a mixture of these sgRNAs led to a similar level of transcriptional activation.

Supplementary Tables

Supplementary Table S1. Percentages of methylated CpG in the four total CpGs in the promoter region of *RANKL* gene.

Group	Left to right (from -700 to the TSS site in the *RANKL* promoter, *ref to* Figure 1e)			
The reference group (no sgRNA guide sequence)	75.0%[a]	100.0%	50.0%	100.0%
RANKL sgRNA-1[b]	37.5%	75%	37.5%	50%
RANKL sgRNA-2	42.9%	71.4%	28.6%	100.0%
RANKL sgRNA-3	18.2%	81.8%	45.5%	81.8%
RANKL sgRNA-4	22.2%	80.0%	40.0%	70.0%
RANKL sgRNA-5	50.0%	86.4%	54.5%	90.9%

a, The methylation percentage was determined by the bisulfite-sequencing method.

b, Totally, five sgRNAs targeting to *RANKL* promoter were assayed.

Supplementary Table S2. Percentages of methylated CpG in eight total CpGs in *MAGEB2* promoter region.

Group	Left to right right (from -700 to -300 prior to the TSS site in the *MAGEB2* promoter, *ref to* Supplementary Figure 1)							
The reference group (no sgRNA guide sequence)	12.0%[a]	16.0%	20.0%	24.0%	24.0%	8.0%	48.0%	76.0%
MAGEB2 sgRNA-2[b]	10.0%	9.5%	14.3%	9.5%	9.5%	4.8%	47.6%	85.7%
MAGEB2 sgRNA-3	10.0%	15.0%	10.0%	10.0%	5.0%	0.0%	50.0%	70.0%
MAGEB2 sgRNA-5	7.7%	12.1%	21.5%	22.5%	26.5%	7.7%	61.3%	77.2%

a, The methylation percentage was determined by the bisulfite-sequencing method.

b, Totally, three sgRNAs targeting to *MAGEB2* promoter were assayed.

Supplementary Table S3. Percentages of methylated CpG in the four total CpGs in the promoter region of *RANKL* gene at different time points post transfection.

Group	Left to right (from -700 to the TSS site in the *RANKL* promoter, *ref to* Figure 1e)			
The reference group (no sgRNA guide sequence)	54.5%[a]	90.9%	45.5%	81.8%
RANKL sgRNA-2 (5 days)	29.4%	88.2%	58.8%	47.1%
The reference group (no sgRNA guide sequence)(7 days)	53.8%	92.3%	46.2%	76.9%
RANKL sgRNA-2 (7 days)	22.2%	77.8%	33.3%	55.6%
The reference group (no sgRNA guide sequence)(9 day)	50.0%	86.4%	54.5%	90.9
RANKL sgRNA-2 (9 days)	13.0%	78.3%	26.1%	69.6%

a, The methylation percentage was determined by the bisulfite-sequencing method.

b, The experiment was independent from that in Figures 1e and 1f, but the results showed a similar trend.

Supplementary Table S4. Comparison of different approaches for DNA demthylation.

Method	Design	Limitation and Advangtage	Refs
ZFN-TET	Composite of ZF motifs that binds to ~3–6 nt triplets of the target DNA; fusion ZF with TET catalytic domain	Engineering challenge, cost high, and potential side effects with over-expression of TET1	4
TALAN-TET	Composite of a series of repeat variable domains that binds to a single nt in the target DNA; fusion TALE with TET catalytic domain	Engineering challenge, cost high, and potential side effects with over-expression of TET1	5
dCas9-TET	Programmable ~20 nt sgRNA to pair with the target DNA; dCas9 fusion with TET catalytic domain	Low cost, easy engineering, scalable for genome screening, and potential side effects with over-expression of TET1	6
CRISPR/dCas9-R2 (this system)	Programmable ~20 nt sgRNA to pair with the target DNA；R2 loop recruit DNMT1 at specific loci and inhibit DNMT1 enzyme activity, break the DNA methylation maintenance	Low cost, easy engineering, and scalable for genome screening	This study

Supplementary Table S5. Primers sequences used in qRT-PCR assay and BSP sequencing.

Gene	Forward primer (5'-3')	Reverse primer (5'-3')
GAPDH	ACCACAGTCCATGCCATCAC	ATGACCTTGCCCACAGCCTT
RANKL-RT	ACTATTAATGCCACCGACA	AGGGTATGAGAACTTGGGAT
MAGEB2-RT	GCTGCGGGTGTTTCATCCA	TGGTTAGAGGATCTTCGCTTGG
RANKL-BSP	ATTTTTTAAATTTTAAGGAGGAAAT	AAAACCAATCAACCCCAAAC
MAGEB2-BSP	GGTTTTAGGTTAAAGAGATAAAATTTA	TAAACCTCACAAACCTAACAACAAC

Supplementary Table S6. Sequences of sgRNA target sites used in the construction of CRISPR/dCas9-R2 expression plasmids.

sgRNA	Target site sequence (PAM region highlighted in red) (5'-3')
RANKL sgRNA-1	TCCTTCATGGGGGCCCTCCAAGG
RANKL sgRNA-2	CAAGGGGAGTCTGGAACCACTGG
RANKL sgRNA-3	ATGGGTTAAAGAAGACGCAGAGG
RANKL sgRNA-4	CTTGAAGGTGACATTGAGCGAGG
RANKL sgRNA-5	CCTCCTCGGATGCTTGCTTCTGG
MAGEB2 sgRNA-1	CTTGGCTTTCACGGGAATCAAGG
MAGEB2 sgRNA-2	TCGCCATTGTTAGCACCGAGAGG
MAGEB2 sgRNA-3	AGAGCCCCCTCGTAACACTTAGG
MAGEB2 sgRNA-4	GGGCCATTTCCACTAGTCCAAGG
MAGEB2 sgRNA-5	CACCAATGCTGTCACCCTTGGGG

References

1 Ran, F. A. *et al.* Genome engineering using the CRISPR-Cas9 system. *Nature Protocols* **8**, 2281, doi:10.1038/nprot.2013.143 https://www.nature.com/articles/nprot.2013.143#supplementary-information (2013).
